# Mutations Found in the *Asc1* Gene That Confer Susceptibility to the AAL-Toxin in Ancestral Tomatoes from Peru and Mexico

**DOI:** 10.3390/plants10010047

**Published:** 2020-12-28

**Authors:** Rin Tsuzuki, Rosa María Cabrera Pintado, Jorge Andrés Biondi Thorndike, Dina Lida Gutiérrez Reynoso, Carlos Alberto Amasifuen Guerra, Juan Carlos Guerrero Abad, Liliana Maria Aragón Caballero, Medali Heidi Huarhua Zaquinaula, Cledy Ureta Sierra, Olenka Ines Alberca Cruz, Milca Gianira Elespuru Suna, Raúl Humberto Blas Sevillano, Ines Carolina Torres Arias, Joel Flores Ticona, Fátima Cáceres de Baldárrago, Enrique Rodoríguez Pérez, Takuo Hozum, Hiroki Saito, Shunsuke Kotera, Yasunori Akagi, Motoichiro Kodama, Ken Komatsu, Tsutomu Arie

**Affiliations:** 1 Bio-Applications and Systems Engineering—BASE, Tokyo University of Agriculture and Technology (TUAT), Fuchu, Tokyo 183-8509, Japan; tsuzukirin@gmail.com; 2 National Institute of Agricultural Innovation (INIA), La Monila 15026, Lima 12, Peru; rcabrera@inia.gob.pe (R.M.C.P.); jbiondith@gmail.com (J.A.B.T.); dgutierrez@inia.gob.pe (D.L.G.R.); camasifuen@inia.gob.pe (C.A.A.G.); jguerreroa@inia.gob.pe (J.C.G.A.); sdb@inia.gob.pe (M.G.E.S.); 3 Plant Pathology Clinic, La Molina National Agrarian University (UNALM), La Monila 15026, Lima 12, Peru; lili@lamolina.edu.pe (L.M.A.C.); medalihuarhua@lamolina.edu.pe (M.H.H.Z.); 20121469@lamolina.edu.pe (C.U.S.); 20170072@lamolina.edu.pe (O.I.A.C.); 4 Crop Husbandry Department, La Molina National Agrarian University (UNALM), La Monila 15026, Lima 12, Peru; rblas@lamolina.edu.pe (R.H.B.S.); 20180830@lamolina.edu.pe (I.C.T.A.); joelflores@lamolina.edu.pe (J.F.T.); 5 Department of Biology, Faculty of Biological Sciences, National University of San Augustín, Santa Catalina, Arequipa 04000, Peru; fatimafloriemay.silva@gmail.com; 6 Department of Plan Breeding, Chapingo Autonomous University, Texcoco, CP 56230, Mexico; erodriguezx@yahoo.com.mx; 7 Interdisciplinary Research Center for Environment and Rural Service, Chapingo Autonomous University, Texcoco, CP 56230, Mexico; taqhoz@hotmail.com; 8 Graduate School of Agriculture, Tokyo University of Agriculture and Technology (TUAT), Fuchu, Tokyo 183-8509, Japan; s180960s@st.go.tuat.ac.jp (H.S.); s197882x@st.go.tuat.ac.jp (S.K.); akomatsu@cc.tuat.ac.jp (K.K.); 9 Tottori BioFrontier, Yonago, Tottori 683-8503, Japan; royaltouch242@gmail.com; 10 Department of Agriculture, Tottori University, Tottori 680-8553, Japan; mk@muses.tottori-u.ac.jp

**Keywords:** *Solanum pimpinellifolium*, *Solanum lycopersicum* var. *cerasiforme*, alternaria alternata tomato pathotype, AAL-toxin, Peru

## Abstract

Tomato susceptibility/resistance to stem canker disease caused by *Alternaria alternata* f. sp. *lycopersici* and its pathogenic factor AAL-toxin is determined by the presence of the *Asc1* gene. Several cultivars of commercial tomato (*Solanum lycopersicum* var. *lycopersicum*, *SLL*) are reported to have a mutation in *Asc1*, resulting in their susceptibility to AAL-toxin. We evaluated 119 ancestral tomato accessions including *S. pimpinellifolium* (*SP*), *S. lycopersicum* var. *cerasiforme* (*SLC*) and *S. lycopersicum* var. *lycopersicum* “jitomate criollo” (*SLJ*) for AAL-toxin susceptibility. Three accessions, *SP* PER018805, *SLC* PER018894, and *SLJ* M5-3, were susceptible to AAL-toxin. *SLC* PER018894 and *SLJ* M5-3 had a two-nucleotide deletion (nt 854_855del) in *Asc1* identical to that found in *SLL* cv. Aichi-first. Another mutation (nt 931_932insT) that may confer AAL-toxin susceptibility was identified in *SP* PER018805. In the phylogenetic tree based on the 18 COSII sequences, a clade (S3) is composed of *SP*, including the AAL-toxin susceptible PER018805, and *SLC*. AAL-toxin susceptible *SLC* PER018894 and *SLJ* M5-3 were in Clade S2 with *SLL* cultivars. As *SLC* is thought to be the ancestor of *SLL*, and *SLJ* is an intermediate tomato between *SLC* and *SLL*, *Asc1*s with/without the mutation seem to have been inherited throughout the history of tomato domestication and breeding.

## 1. Introduction

Agricultural plant evolution has been driven by a complex process involving human activities and natural environment. Humans have selected individual wild plants displaying preferable traits, for example suitable for eating, resulting in domestication of plants [1]. Modern plant breeding has enhanced the selection of genes determining favorable phenotypes within a diverse gene pool, which has led to a reduction in genetic diversity among agricultural plants.

Tomato (*Solanum lycopersicum* L., formerly *Lycopersicon esculentum* Mill; *SLL*) is the most abundantly produced vegetable in the world. The total production of tomatoes was ca. 0.2 billion tons from ca. 5 million ha of fields in 2019 [2]. *SLL* originated from *S. pimpinellifolium* L. (*SP*) in the Andean region of South America, now occupied by Peru, Chile, Ecuador, and Bolivia [3,4,5,6]. The history of tomato domestication began about 2000 years ago, possibly in Mexico; subsequently, tomato was brought to Europe around 500 years ago [6,7,8]. The Andes region continues to sustain wild tomato species, including not only *SP* but *S. chilense* (Dunal) Reiche, *S. chmielewskii* Rick, *S. habrochaites* Knapp et Spooner, *S. neorickii* Spooner et al., *S. pennellii* Correll, and *S. peruvianum* L. [3,4,5,6]. *S. lycopersicum* var. *cerasiforme* (Dunal) A.Gray (*SLC*), an apparent intermediate hybrid between *SP* and *SLL*, is currently found as a native-grown tomato in Mexico and several Central and South American countries, such as Peru, Chile, Ecuador, Bolivia, and Columbia [5,9,10]. Traditional *SLL* cultivars, considered to be the archetype of modern *SLL* cultivars, have been handed down by generations of peasants in mountain villages in Mexico and designated “jitomate criollo” in Spanish (*SLJ*) [5,9]. *SLC* and *SLJ* are sometimes collectively called transition tomatoes [5].

*Solanum* fruits have diverse colors. Species with orange and red fruit are in the Lycopersicon species group [11] and include *SP*, *SLC*, *SLL*, *S. cheesmaniae* (*SC*) and *S. galapagense* (*SG*), the latter two of which are found in the Galápagos Islands, Ecuador. A previous study proposed that the Lycopersicon species group originated from red-fruited *SP*; initially *SP* was domesticated in South America to give rise to *SLC*, and *SLC* later gave rise to *SLL* in Mesoamerica through subsequent selection and breeding [4,10].

A phylogenetic study reported that Peruvian *SP* and/or *SLC* were transported by the Humboldt Current or the Peru Current to the Galápagos Islands where they settled and established *SC* and *SG* [11]. Interestingly, there are collection reports of finding *SLC* in the Hawaii Islands, the Philippines, and Malaysia [12] suggesting that tomato seeds can be carried long distances by ocean currents.

Alternaria stem canker disease caused by the ascomycete fungus *Alternaria alternata* forma specialis (f. sp.) *lycopersici* (or, *A. alternata* tomato pathotype; *Aal*) is an important disease in tomatoes. In 1975, the disease was reported for the first time in the *SLL* cultivar (cv.) Earlypak 7 in California, USA [13], followed by a 1977 report of the pathogen infecting cv. Aichi-first in Japan [14]. Most of the other *SLL* cultivars are resistant to the disease [13,15]. Purified AAL-toxin produced by *Aal*, a host-specific toxin, is toxic only to those cultivars susceptible to *Aal* and causes necrotic lesions but not in the cultivars resistant to *Aal* [16]. Among wild tomatoes, *SC* and *SG* from the Galápagos Islands are known to be susceptible to the AAL-toxin [17]. 

AAL-toxin is the leading cause of symptom development in stem canker disease [18]. AAL-toxin induces apoptotic cell death in *SLL* tissues; however, cultivars resistant to AAL-toxin produce ceramide that protects the tissues from cell death [18]. The *Asc1* (alternaria stem canker resistance protein 1) gene encodes an enzyme involved in ceramide biosynthesis in *SLL* [18]. The *SLL* cv. Aichi-first, which is susceptible to AAL-toxin, has a two-nucleotide deletion in the *Asc1* ORF, and *SC* and *SG* have ca. a 400 nucleotide-deletion that includes the 5′-UTR and a part of the 5′ ORF of *Asc1* [17]. 

We hypothesized that the mutations found in *Asc1* in the AAL-toxin susceptible cultivars and *SC* and *SG* originated from the gene pool of *Asc1* in *SP* and *SLC*, the possible wild ancestors of *SC* and *SG*, and that we could find variations of *Asc1* mutations in *SP* and *SLC*. To test this hypothesis, we established a collection of *SP* in Peru and Ecuador; *SLC* in Peru, Ecuador and Mexico; and *SLJ*, the archetypes of *SLL,* in Mexico. We investigated their susceptibilities to AAL-toxin and determined the nucleotide sequences of the respective *Asc1* genes. 

## 2. Results

### 2.1. UNALM-TUAT Collection of Peruvian Tomatoes

**Figure 1:**
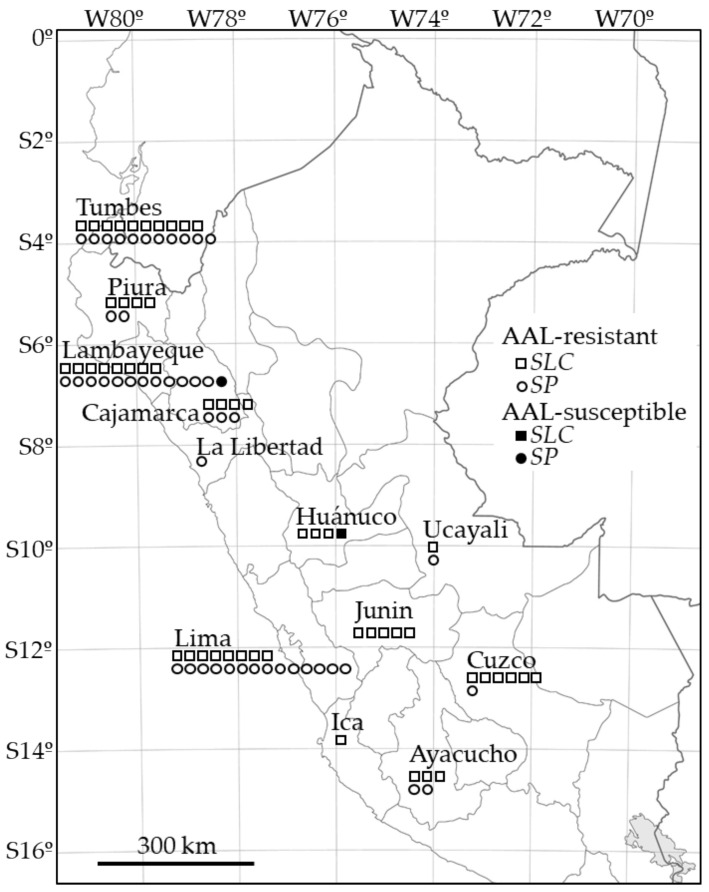
Map of the collection sites of Peruvian tomato accessions. Squares shown within each province represent accessions of *Solanum lycopersicum* var. *cerasiforme* (*SLC*) and circles represent *S. pimpinellifolium* (*SP*) from the UNALM-TUAT Collection and the INIA Collection (Table 1). Each black square and circle shows an AAL-toxin susceptible accession. Map from Aflo Co. [19] and modified.

From 2016 to 2019, we collected wild tomatoes throughout several field trips in Peru and created the UNALM-TUAT Collection of Peruvian tomatoes composed of 41 *SLC* and 19 *SP* accessions (Table 1). In order to construct a diverse collection of wild tomatoes, we collected throughout a large area of Peru that encompassed the northwestern coast area including Tumbes, Piura, Lambayeque and La Libertad Regions, the northern highland and semi-jungle area, including the Cajamarca and Huánuco Regions, the Amazon rainforest area including the Ucayali Region, the south-central highland area including the Junin, Cusco and Ayacucho Regions, and the Pacific coastal area including the Lima and Ica Regions. Usually *SP* and *SLC* are found in coastal areas that are not over 800 m in elevation, but we also found *SP* and *SLC* in valleys in the Andean Mountains like Quillabamba City in the Cusco Region. *SP* and *SLC* were not distributed in the untouched natural environments but rather in fallow agriculture fields and near inhabited centers. Figure 1 schematically presents the sampling areas for *SLC* (squares) and *SP* (circles) in the UNALM-TUAT and INIA Collections used in this study. 

### 2.2. Accessions Susceptible to AAL-Toxin

**Figure 2:**
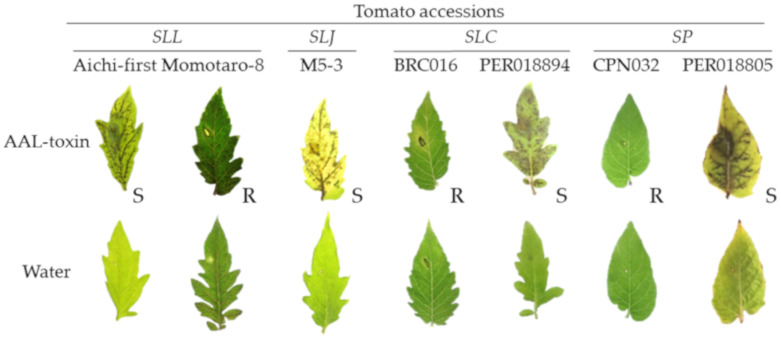
Leaflet bioassay for AAL-toxin from a culture extract of *Aal* As-27. The abaxial side of each tomato leaflet was wounded and a small piece of filter paper containing either the culture extract or water was placed on the wound and incubated in a humidified chamber (25 °C for 3 days). The necrosis of the leaflet was evaluated, and a susceptible reaction or resistant reaction is indicated with an “S” or “R”, respectively, in the figure. *SLL* cv. Aichi-first is a representative cultivar with susceptibility to AAL-toxin. *SLL* cv. Momotaro-8 is a representative cultivar with resistance to AAL-toxin. M5-3 was susceptible to AAL-toxin among two accessions of *SLJ*. Among 60 accessions of *SLC*, one accession, PER018894, was susceptible to AAL-toxin, and the others were resistant to AAL-toxin. The reaction of BRC016 is representative of AAL-resistant *SLC* accessions. Among 37 accessions of *SP* one accession, PER018805, was susceptible to AAL-toxin, and the others were resistant to AAL-toxin. CPN032 is representative of AAL-resistant *SP* accessions.

In bioassays using leaflets, one (M5-3 sampled in Querétaro, Mexico) among the two *SLJ* accessions, one (PER018894 from Huanuco, Peru) among the 62 *SLC* accessions, and one (PER018805 from Lambayeque, Peru) among the 51 *SP* accessions presented veinal necrosis and were determined to be susceptible to AAL-toxin (Table 1 and Figure 2). Other accessions presented no symptoms (Table 1 and Figure S2), suggesting that they are resistant to AAL-toxin. The references, *SC* (LA 0437 and 0521), *SG* (LA 0438 and 0528) and *SLL* cv. Aichi-first were susceptible to AAL-toxin, and *SLL* cv. Momotaro-8 was resistant to AAL-toxin.

### 2.3. Absence of ca. 400-bp Deletion in Asc1 in SP, SLC and SLJ

**Figure 3:**
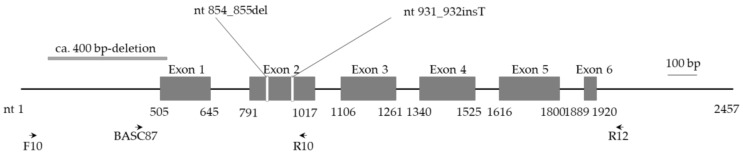
Schematic structure of *Asc1* from *SLL* in the DDBJ/EMBL/GenBank databases identified as accession #AF198177. *Asc1* is composed of 6 exons and encodes an ASC1 protein of 308 amino acids. Primers indicated by arrows are listed in Table 2. The primer set BASC87+R12 was used to amplify a ca. 1600-bp fragment containing *Asc1* for cloning and sequencing. The primer set F10+R10 was used to detect the ca. 400-bp deletion including the 5′-UTR and part of the 5′ ORF of *Asc1*. White gaps shown in exon 2 represent a two-nucleotide deletion reported in *SLL* cv. Aichi-first and found in *SLJ* PER018894 and *SLC* M5-3 in this study and a nucleotide insertion found in *SP* PER018805, respectively. An approximately 400-bp deletion including the upstream region and part of the 5′ ORF region indicated by a gray bar has been reported in *SC* and *SG* [17].

**Figure 4:**
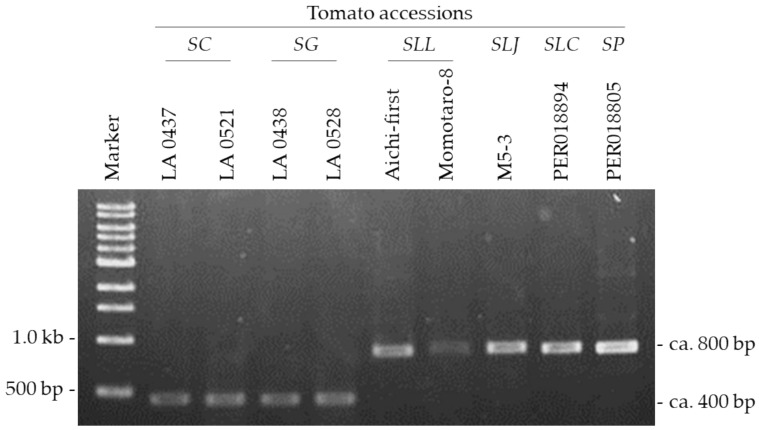
PCR amplification with the primer set F10+R10 (Table 2) to detect the ca. 400-bp deletion including the 5′-UTR and part of the 5′ ORF of Asc1 (Figure 3). Only the reference accessions of *SC* and *SG* had the ca. 400 bp-deletion and none of the tested *SP*, *SLC*, and *SLJ* accessions had the deletion. In this figure, only representative accessions of *SP*, *SLC*, and *SLJ* are presented. Marker, 1 kb DNA Ladder (New England Biolabs, Ipswich, MA, USA).

The susceptibility to AAL-toxin in *SC* and *SG* is determined by a ca. 400-bp deletion that includes the 5′-UTR and part of the 5′ ORF of *Asc1* (Figure 3) [17]. PCR using a F10/R10 primer set reveals that all tested accessions, including the three AAL-toxin susceptible accessions (*SLJ* M5-3, *SLC* PER018894 and *SP* PER018805), did not have the ca. 400-bp deletion in the *Asc1* region (Table 1 and Figure 4). The references *SC* (LA 0437 and LA 0521) and *SG* (LA 0438 and LA 0528) had the ca. 400-bp deletion as previously reported [15].

### 2.4. Mutations in Asc1

We sequenced the all of the *Asc1* region of the tomato accessions except for 39 of the accessions from the INIA Collection and compared the sequences with that of the reference AAL-resistant *SLL* (Acc. #AF198177) [20,21,22,23]. 

**Figure 5:**
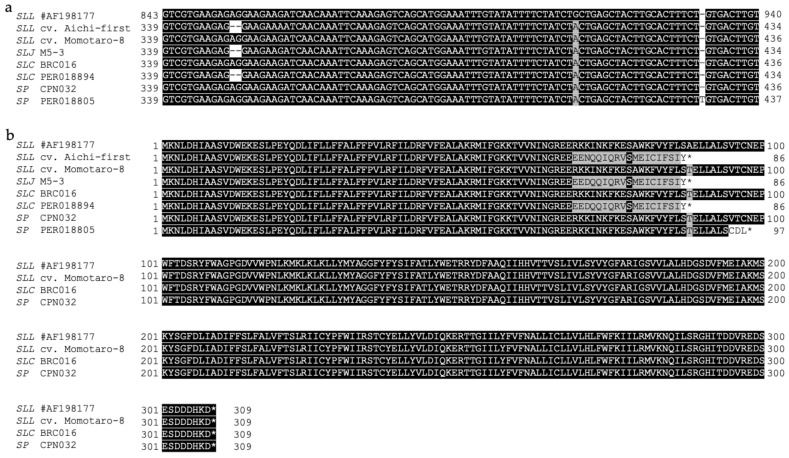
(**a**) Nucleotide variations found in exon 2 of *Asc1* (Figure 3). Identical nucleotides are highlighted in black in comparison to the reference sequence of *SLL* #AF198177 (resistant to AAL-toxin). *SLL* cv. Aichi-first (susceptible), *SLJ* M5-3 (susceptible) from Mexico and *SLC* PER018894 (susceptible) from Peru have the nt 854_855del mutation, and *SP* PER018805 (susceptible) from Peru has the nt 931_932insT mutation. (**b**) Deduced amino acid sequences of *Asc1*. The amino acid sequences were aligned using CLUSTALW [24]. Identical and similar amino acids are highlighted in black or gray, respectively, by GeneDoc [25]. * indicates termination. In comparison to the reference sequence of *SLL* #AF198177 (resistant to AAL-toxin), *SLL* cv. Aichi-first (susceptible), *SLJ* M5-3 (susceptible), *SLC* PER018894 (susceptible) and *SP* PER018805 (susceptible) produce smaller proteins that may be nonfunctional.

*Asc1* sequences of *SLJ* M5-3 and *SLC* PER018894, both of which were susceptible to AAL-toxin by the leaflet test, had the two-nucleotide deletion (nt 854_855del) in the second exon and generated a frameshift and possibly produced a non-functional protein (Figure 5 and Table 1). This two-bp deletion was identical with that reported for *SLL* cv. Aichi-first, an AAL-toxin susceptible cultivar [17]. 

*SP* PER018805, susceptible to AAL-toxin by the leaflet test, had a T-insertion (nt 931_932insT) in the second exon of *Asc1*, causing a frameshift that might generate a smaller, premature asc1 protein (Figure 5 and Table 1). This mutation in the *Asc1* gene has not been reported previously. Although involvement of this mutation in AAL toxin-susceptibility in PER018805 can be genetically confirmed by outcrossing PER018805 with an AAL-resistant *SP* accession, the regulation of studies on wild tomatoes in Peru has prevented this experiment from being conducted. 

Only five Mexican *SLC* accessions (M-UX, MC-5a, MC-5b and ML-1 in the TUAT Collection and LA 1623 in the TGRC Collection) had an *Asc1* DNA sequence identical with #AF198177. 

We found eleven kinds of missense mutations (509A>G, 569A>C, 570G>A, 572A>G, 617G>A, 807T>C, 836A>T, 911G>A, 1010A>C, 1366T>C, 1693T>G) in the *Asc1* sequence in 31 accessions (Table 1). Many silent mutations were also detected in *Asc1* nucleotide sequences of these accessions (Table 1).

### 2.5. Phylogeny

**Figure 6:**
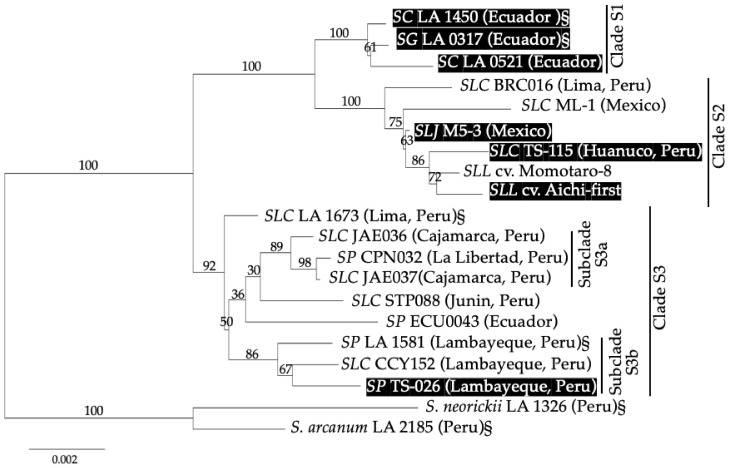
A maximum likelihood (ML) tree based on 18 COSII sequences of tomato accessions estimated using Modeltest-NG ver. 0. 1. 6 [26] and RAxML-NG v. 1.0.0 [27]. *S. arcanum* and *S. neorickii* were used as outgroups. The bootstrap values were calculated after 1,000 bootstrap replicates. The data sets of *SG* (LA 0317), *SC* (LA 1450), *SLC* (LA 1673), *SP* LA 1581), *S. arcanum* (LA 2185) and *S. neorickii* (LA 1326) are from [10] and are indicated with a § symbol in the tree. AAL-toxin susceptible accessions are highlighted with a black background. *SC*, *S. cheesmaniae*; *SG*, *S. galapagense*; *SLC*, *S. lycopersicum* var. *cerasiforme*; *SLJ*, *S. lycopersicum* var. *lycopersicum* “jitomate criollo”, *SLL*, *S. lycopersicum* var. *lycopersicum* (commercial varieties); *SP*, *S. pimpinellifolium*.

The maximum likelihood (ML) phylogeny tree based on 18 COSII sequences is presented in Figure 6. The tree formed three clades supported by high bootstrap values, designated in this study as S1, S2 and S3. Clade S1 is composed only of Galápagos tomatoes, including *SC* and *SG*. Clade S2 is composed of *SLL* commercial cultivars, *SLJ* and *SLC* from Mexico and Peru. Clade S3 is composed of *SLC* and *SP* from Peru and Ecuador only. All of the tested *SP* accessions were in Clade S3.

The accessions *SLJ* M5-3 and *SLC* PER018894, susceptible to AAL-toxin and carrying the identical mutation (nt 854_855del) in *Asc1* as *SLL* cv. Aichi-first, were in Clade S2 with *SLL* cv. Aichi-first. The *SP* accession, PER018805, susceptible to AAL-toxin and with a mutation (nt 931_932insT) in *Asc1,* was placed in Clade S3. 

The topology of the ML tree did not contradict that of the BI tree (Figure S3).

## 3. Discussion

In this study, we evaluated 119 ancestral tomato accessions for their susceptibility to AAL-toxin produced by *Aal*. Only three accessions, an *SLJ* from Mexico, an *SLC* from Peru and an *SP* from Peru, were susceptible to AAL-toxin; the others were resistant. The number of AAL-toxin susceptible accessions was less than expected. This is the first time that AAL-toxin susceptible *SLJ* and *SLC* have been reported.

Among the three AAL-toxin susceptible accessions, *SLJ* M5-3 sampled from Mexico and *SLC* PER018894 from Peru had a frameshift mutation (nt 854_855del) identical to that found in *SLL* cv. Aichi-first, also an AAL-toxin susceptible accession. As *SLC* is thought to be the oldest progenitor of present-day commercial cultivars and *SLJ* is an intermediate tomato between *SLC* and present-day commercial *SLL*, both of *Asc1* genes with the frameshift mutation (nt 854_855del) and without the frameshift seemed to have been passed down throughout the history of tomato domestication and modern breeding.

As *SP* and its derivative species, *SC* and *SG*, have been collected from the Galápagos Islands and the Hawaiian Islands [11], it has been proposed that *SP* seeds were carried to the islands from the South American mainland by the Humboldt Current. Interestingly, all *SC* and *SG* accessions from the Galápagos Islands evaluated so far are AAL-toxin susceptible and have a ca. 400-bp deletion in *Asc1* [17]. We inferred that the genetic diversity of *SP*, including the *Asc1* gene, is rich in areas considered to be the center of origin of this species. One of the strains having the ca. 400-bp deletion in *Asc1* was carried to Galápagos Islands by the Humboldt Current to establish *SC* and *SG* there. We also hypothesized that the original *SP* strains having the ca. 400-bp deletion in *Asc1* still survive in South America, the proposed center for the origin of tomatoes. Therefore, we sequenced *Asc1* from 23 *SP* accessions (Table 1). Contrary to our expectations, no *SP* accession with ca. 400-bp deletion has been found. It is possible that we have not yet identified the place of origin of the *SP* that crossed the ocean to the Galápagos Islands. 

Although the diversity of *Asc1* among the accessions seemed not as rich as expected (Table 1), we found that PER018805, one of the *SP* accessions from Lambayeque in Northwestern Peru, had a frameshift mutation, nt 931_932insT, in the second exon of *Asc1* (Table 1). This mutation generates the production of a smaller (97 aa.) and possibly premature asc1 protein (Figure 5) and is reported here first. 

Sequencing of *Asc1* identified the frequent presence of missense mutations (509A>G, 569A>C, 570G>A, 572A>G, 617G>A, 807T>C, 836A>T, 911G>A, 1010A>C, 1366T>C, 1693T>G) that did not affect the susceptibility to AAL-toxin (Table 1). 

Since the stem canker disease pathogen *Aal* has not been reported in South America, susceptibility/resistance to *Aal* or to AAL-toxin may not be a factor in the selection of *Asc1* mutations. These findings suggested that if we analyze more accessions of *SP*, we may find accessions having more diverse *Asc1* sequences. 

Silent mutations were frequently detected in introns and exons. Especially 1306T>G in the third intron was common in *SLJ* (2 among the 2 accessions sequenced), *SLC* (46 among 51), *SP* (22 among 23) and both of the *SLL* commercial varieties (Table 1), suggesting that the *Asc1* sequence of *SLL* #AF198177 used as a reference in this study was not an ideal standard type. 

From 2000 to 2019, we tried to isolate *Alternaria* spp. from the tissues of ancestry tomato accessions in Chile, Ecuador, Mexico and Peru. We examined *SP*, *SLC*, *SLJ*, *SLL*, *S. chilense*, *S. peruvianum*, *S. penellii* and samples of the surrounding air and soil. Although we obtained hundreds of *Alternaria* spp., no isolate causing stem canker in tomato was found (data not shown) [28]. We have studied the co-evolution of tomato and tomato wilt pathogen, *F. oxysporum* f. sp. *lycopersici* [3,5]. Tomato and the stem canker pathogen *Aal* seem also likely to be a good model system for co-evolution analysis.

The ML phylogeny tree (Figure 6) formed three clades (S1–S3). Galápagos tomatoes, *SC* and *SG*, all of which are susceptible to AAL-toxin and have the ca. 400-bp deletion in *Asc1*, were grouped together as Clade S1, in agreement with a previous report [11]. 

Clade S2 is composed of *SLC* from Mexico and Peru, *SLJ* from Mexico and *SLL* commercial cultivars (Figure 6). Although the number of accessions tested in this study is small, clade S2 seems to support the hypothesis that the present commercial tomato (*SLL*) was established from *SLC* via *SLJ.* Our finding was consistent with the report by Raziferd et al. (2020) [10]. Clade S2 includes AAL-susceptible *SLC* (PER018894), *SLJ* (M5-3) and *SLL* cv. Aichi-first, AAL-resistant *SLC* (BRC016 and ML-1) and *SLL* cv. Momotaro-8. All three of the AAL-susceptible accessions had the identical frameshift mutation (nt 854_855del) in *Asc1*, which again suggested that *Asc1* with and without the nt 854_855del frameshift mutation have been passed down throughout the history of tomato domestication and modern breeding from *SLC* to *SLL*. The mutation was found only in clade S2. 

All of the tested *SP* accessions from Peru and Ecuador were grouped in Clade S3. Clade S3 also includes *SLC* accessions from Peru and Ecuador. Identification of *SP* and *SLC* in this study was based on the morphological characteristics first detailed by Darwin et al. (2003) [9]. *SP* and *SLC* are often very similar in morphology, and there have been many discussions on how to classify them correctly [9,10,11]. Our phylogeny based on the COSII complex region again indicated that *SP* and *SLC* are genetically indistinguishable (Figure 6). JAE036, JAE037 and CPN032 constituted a subclade (S3a) supported with a bootstrap value of 89. The accessions LA1581, CCY152, and PER018805, all of which were collected in Lambayeque Province in different years, constituted another subgroup (S3b) with a boot strap value of 86 (Figure 1 and Figure 6). Interestingly one of the accessions, PER018805 was susceptible to AAL-toxin and had a newly identified mutation (nt 931_932insT) in *Asc1*. From the phylogenetic tree, this mutation appears to have occurred independently within this subclade. 

Most of the accessions in Clade S3 were collected from Cajamarca, La Libertad and Lambayeque Regions, which are geographically close, suggesting that this northwestern area might be the center of origin for tomatoes. Moreover, the two Peruvian *SLC* accessions, BRC016 and PER018894, in Clade S2 were collected in Lima and Huanuco Provinces, respectively, both of which are in central Peru. These results suggest that these *SLC*s had already formed an evolutionary branch to *SLL*, and, moreover, the *SLC* in central Peru was likely the germplasm brought to Mesoamerica. 

## 4. Materials and Methods

### 4.1. Plant Materials

The *Solanum* accessions used in this study are listed in Table 1. From the TUAT Collection (Laboratory of Plant Pathology, Tokyo University of Agriculture and Technology (TUAT), Fuchu, Tokyo) [5], two *SLJ* and four *SLC* accessions from Mexico and two *SLC* and two *SP* accessions from Ecuador were used. No *SP* or *SLJ* accessions were collected in Mexico or Ecuador, respectively.

The UNALM-TUAT Collection (La Molina, Peru) of Peruvian tomatoes is composed of 41 *SLC* and 19 *SP* accessions sampled from 2016 to 2019. Details about this collection are described in the Results section.

From the INIA Collection (La Molina, Lima, Peru) 12 *SLC* and 29 *SP* accessions sampled from Ayacucho, Cajamarca, Cusco, Huanuco, Lambayeque, Lima, and Ucayali regions of Peru were used (Table 1). 

*SC* (LA 0437 and LA 0521) and *SG* (LA 0438 and LA 0528), which are susceptible to *Aal* and its culture extract that contains AAL-toxin, were obtained from the TGRC Collection, C.M. Rick Tomato Genetics Resource Center (Davis, CA, USA). Three additional *SLC* accessions (LA 1456, LA 1632 and LA 1909) and one *SP* (LA 3123) accession from the TGRC Collection were used as references.

Cultivated tomato, *SLL* cvs. Momotaro-8 (Takii & Co., Kyoto, Japan) and Aichi-first (Matsunaga Seed, Konan, Aichi, Japan) were also used as references. Momotaro-8 is a cultivar that is resistant to *Aal* and to its culture extract containing the AAL-toxin. In contrast, cv. Aichi-first is susceptible to *Aal* and the culture extract (Figure S1) [14]. 

For the accessions from the TUAT, TUAT-UNALM and TGRC Collections and the commercial cultivars, three to five seeds were sown in sterilized soil (Nippi Engei Baido; Nihon Hiryo Co, Chuo, Tokyo, Japan) in plastic pots (7 cm in diameter) and were grown in a greenhouse maintained at around 28 °C for about three weeks. Leaflets (or folioles) were harvested. For the accessions in the INIA Collection, leaflets were harvested from plants grown in a greenhouse for about three weeks at INIA (La Molina. Peru) and the INIA Donoso Agriculture Experiment Station (Huaral, Peru). 

### 4.2. Fungal Isolate and the Preparation of Culture Extracts Containing the AAL-Toxin

*Alternaria alternata* f. sp. *lycopersici* As-27 (*Aal*) maintained in the Laboratory of Plant Pathology, Tottori University, Tottori, Japan was used in this study [29,30]. The isolate is the pathogen responsible for tomato stem canker disease and also produces AAL-toxin [22]. The isolate was maintained on V-8 juice agar medium [31] in the dark at 28 ºC and was used to prepare culture extracts.

Culture extracts of *Aal* containing the AAL-toxin were prepared following a published protocol [32] with a slight modification. Briefly, *Aal* was cultured in a modified Richard’s liquid medium (1 L) at room temperature for two weeks. The mycelium was removed by filtration using filter paper (No. 1, Toyo Roshi Kaisha, Chiyoda, Tokyo, Japan), and the filtrate was lyophilized using a freeze-dryer (VD-500, TAITEC Co., Koshigaya, Saitama, Japan), dissolved into 100 mL 70% (*v*/*v*) acetonitrile and used as the *Aal* culture extract containing the AAL-toxin. We assessed the presence of the toxin by bioassay using cv. Aichi-first by the same manner described in 4.3.

### 4.3. AAL-Toxin Susceptibility Assay

The test was conducted following previously reported procedures with a slight modification [30,32,33]. Briefly, a droplet (3 µL) of the *Aal* culture extract was pipetted onto a 3 mm square filter paper (No. 2, Toyo Roshi Kaisha) and air dried to vaporize acetonitrile. Three-week old tomato leaflets were detached, and the abaxial side of each leaflet was wounded slightly by rubbing with a paper towel. A droplet (30 µL) of sterilized distilled water was applied to the leaflet wound, and the filter paper containing the culture extract was placed on the water droplet. Filter paper to which a droplet (3 µL) of sterile distilled water (SDW) had been applied was used as the control. The treated leaflets were placed in a humid square petri dish (140 × 100 × 14.5 mm, Eiken Chemical, Taito, Tokyo, Japan) and maintained at 25 °C for three days. Development of veinal necrosis on the leaflet was evaluated using *SLL* cv. Aichi-first (susceptible to AAL-toxin and presenting veinal necrosis) and cv. Momotaro-8 (resistant to AAL-toxin and presenting no symptoms) as positive and negative controls, respectively. 

To conserve genetic resources, wild tomato seeds cannot be transported from Peru, and, moreover, *Aal*, the stem canker pathogen that has not invaded Peru, could not be transported into Peru; thus, we have not conducted *Aal*-inoculation tests using wild tomatoes.

### 4.4. Tomato Genomic DNA Extraction

Genomic DNA from each tomato accession was purified from leaflets by a cetyltrimethylammonium bromide (CTAB) protocol [34]. Freeze-dried leaflets were powdered using a mortar and pestle and dissolved in 700 µL of CTAB buffer (2.0% (*w*/*v*) CTAB, 0.1 M Tris-HCl pH 8.0, 0.02 M EDTA pH 8.0, and 8.2% (*w*/*v*) NaCl in Milli-Q water) containing 0.5% (*v*/*v*) β-mercaptoethanol, and incubated at 65 °C for 45 min with occasional mixing by gentle swirling. To each tube, an aliquot (700 µL) of chloroform:isoamyl alcohol=24:1 (v:v) (CIA) was added, mixed by inversion to form an emulsion, and centrifuged at 10,000× *g* for 10 min at room temperature. The aqueous phase was harvested and added to 60 µL of 10× CTAB buffer. After mixing, the samples were again extracted with CIA (700 µL), mixed by inversion, and centrifuged in the same conditions. The aqueous phase was combined with isopropanol (500 µL), mixed well to precipitate DNA and centrifuged for 30 min at room temperature. After centrifugation the supernatant layer was removed carefully, and the precipitated DNA was twice washed with 99% ethanol (500 µL). The DNA pellet was air-dried and dissolved in 50 µL of Milli-Q water. 

### 4.5. PCR

The reference nucleotide sequence of *SLL Asc1* is archived in the GenBank database under accession #AF198177. The *SLL Asc1* gene is composed of 6 exons (nts 505–645, 791–1017, 1106–1261, 1340–1525, 1616–1800, and 1889–1920) that encode a protein composed of 308 amino acids. In this report the nucleotide positions are assigned in reference to this accession unless otherwise stated.

Primer set BASC87/R12 (Table 2 and Figure 3) was used to amplify a fragment of ca. 1600 bp encoding *Asc1*. The reaction mixture (10 µL) contained 40 ng of gDNA, 0.4 nmol of each primer, 1× Buffer (Toyobo, Osaka, Japan), 0.2 nmol each dNTP (Toyobo) and 0.2 U of KOD plus NEO polymerase (Toyobo). The thermal conditions are presented in Table 2.

To detect a specific deletion of ca. 400 bp that includes the 5′ UTR and part of the 5′ ORF of *Asc1* as found in *SC* and *SG* [17], the primer set F10/R10 (Table 2 and Figure 3) was used. The reaction mixture (10 µL) contained 40 ng of gDNA, 0.3 µM each primer, 1× Ex-*Taq* buffer (Takara Bio, Kusatsu, Shiga, Japan), 200 µM each dNTP, and 0.25 U of Ex-*Taq* polymerase (Takara Bio). 

The amplicons were separated in a 1% (*w*/*v*) agarose gel by electrophoresis using TAE buffer and were visualized by staining with 0.5 µg/mL ethidium bromide. 

### 4.6. DNA Sequencing

Amplicons obtained with the primer set BASC87/R12 were purified using ExoSAP-IT (Thermo Fisher Scientific, Santa Clara, CA, US), attached to a fluorescent dye by STeP PCR [35], and sequenced with an ABI 3130xl Genetic Analyzer (Thermo Fisher Scientific) using a BigDye Terminator v3.1 Cycle Sequencing Kit (Thermo Fisher Scientific). For each accession three individual PCR reactions and three times sequencing for each reaction by both directions were performed. When the sequences obtained were not identical, we performed additional PCR/sequencing and the sequence was finalized by “majority vote”.

The obtained sequences of *Asc1* were aligned with that of #AF198177 (2457 bp) as the reference sequence using Molecular Evolutionary Genetics Analysis Version 7.0 for Bigger Datasets MEGA7 [36] and GeneStudio.exe [37]. Deduced amino acid sequences were obtained using EMBOSS Six pack [38]. 

### 4.7. Phylogenetic Analysis of Tomato Accessions

The phylogenetic relationships among the 14 tomato accessions (indicated with an asterisk in Table 1) and the reference accessions were analyzed based on their conserved orthologous set (COSII) of nuclear loci [11]. Eighteen COSII markers for each accession were amplified by PCR using the primer sets, sequenced, and combined [11]. Details about the primers and PCR conditions are described in Table S1. The combined sequences of the tested accessions and the reference sequences of six *Solanum* spp., including *SC*, *SG*, *SLC*, *SP*, *S. arcanum*, and *S. neorickii* in the GenBank databases (Table S2), were subjected to phylogenetic analyses using MEGA7 and MAFFT version 7 [39] (https://mafft.cbrc.jp/alignment/server/index.html, accessed on September 16, 2020). All gaps in the alignment were ignored in the following analyses. The phylogenies were estimated using two methods including maximum likelihood ML [40] and Bayesian inference (BI) [41]. The data obtained for *S. arcanum* (LA 2185) and *S. neorickii* (LA 1326), both of which are accessions in the TGRC Collection, were used as the outgroups [11]. 

ML analysis was evaluated with Modeltest-NG ver. 0. 1. 6 [26] using Akaike Information Criterion (AIC). ML phylogeny was estimated using RAxML-NG v. 1.0.0 [27] that allows each partition (each COSII) to have its own model and parameters. Modeltest-NG determined the appropriate substitution model for each respective COSII region (Table S3). To evaluate the stability of the clade on the optimal tree, a bootstrap analysis was performed with 1000 bootstrap replicates. Each branch was statistically estimated by a bootstrap (BS) test in ML analysis and posterior probability (PP) in BI analysis. 

BI phylogenetic analysis also was performed using MrBayes version 3. 2. 7a [42]. Model parameters for DNA data were chosen according to the criteria described above. Tree searching using MrBayes was performed for 1,000,000 generations with trees sampled every 100 generations. A conservative burn-in period was determined, and only post burn-in trees were saved. Finally, the posterior probabilities of each branch were calculated. 

## 5. Conclusions

AAL toxin- susceptible *SP* and *SLC* were found in this study for the first time, and that the nt 931_932insT mutation found in *SP* may confer AAL-toxin susceptibility is the novel report.

Moreover, in Clade S2, we found two AAL-toxin susceptible accessions (*SLC* PER018894 and *SLJ* M5-3) that had the nt 854_855del mutation in *Asc1*. The mutation was identical to that of cv. Aichi-first, an AAL-toxin susceptible commercial cultivar of *SLL*. This finding suggested that this deletion mutation in *Asc1* might have passed down throughout the history of tomato domestication and modern breeding from *SLC* to *SLL*.

Since plant breeding is usually carried out by crossing with wild species, conserving the rich genetic resources of wild species is an important issue. We suggest that several wild tomato genetic resources have influenced the transition and breeding of tomatoes so far and that rich genetic resources will continue to play an important role in the future breeding of this globally important crop.

## Figures and Tables

**Table 1:** Tomato accessions and cultivars used in this study, their susceptibility to AAL-toxin and mutations found in *Asc1.*

Species and Accessions		Sampling Site					AAL-Toxin Susceptibility	Gen Bank Accession No.	Mutations in *Asc1* in Comparison to the Reference Sequence #AF198177 ^b^				
									ca. 400 bp-Deletion ^c^	Frameshift Mutation	Missense Mutation	Silent Mutation	
		Country	Region	Longitude	Latitude	Date ^a^						Exon	Intron
TUAT Collection (Inami et al.2014)													
*S. lycopersicum* var. *lycopersicum* “jitomate criollo” (*SLJ*)													
*	M5-3	Mexico	Queretaro	N21°16′00″	W99°24′20″	20100530	Susceptible	LC596579	No deletion	854_855del	911G>A		1065G>A, 1306T>G
	M5-4	Mexico	Queretaro	N21°16′00″	W99°24′20″	20100530	Resistant	LC596581	No deletion		911G>A		1065G>A, 1306T>G
*S. lycopersicum* var. *cerasiforme* (*SLC*)													
	M-UX	Mexico	Yucatan	N20°24′35″	W89°45′04″	20051229	Resistant	LC596506	No deletion				
	E0040W	Ecuador	Santa Cruz	S00°39′06″	W90°24′21″	20080113	Resistant	LC596555	No deletion		911G>A, 1010A>C		1306T>G
	E0043	Ecuador	Santa Cruz	S00°41′45″	W90°19′36″	20080113	Resistant	LC596554	No deletion		911G>A, 1010A>C		1306T>G
	MC-5a	Mexico	Hidalgo	N21°00′08″	W98°32′18″	20100528	Resistant	LC596505	No deletion				
	MC-5b	Mexico	Hidalgo	N21°00′08″	W98°32′18″	20100528	Resistant	LC596504	No deletion				
*	ML-1	Mexico	Hidalgo	N21°01′06″	W98°31′46″	20100529	Resistant	LC596503	No deletion				
*S. pimpinellifoloum* (*SP*)													
*	ECU0043	Ecuador	Santa Cruz	S00°41′23″	W90°19′10″	20080113	Resistant	LC596553	No deletion		911G>A, 1010A>C		1306T>G
	ECU0045	Ecuador	Santa Cruz	S00°40′05″	W90°16′08″	20080113	Resistant	LC596552	No deletion		911G>A, 1010A>C		1306T>G
UNALM-TUAT Collection													
*S. lycopersicum* var. *cerasiforme* (*SLC*)													
*	BRC016	Peru	Lima	S12°08′21″	W77°01′35″	20161104	Resistant	LC596570	No deletion		911G>A		1306T>G
	CCY138	Peru	Lambayeque	S06°44′06″	W79°32′46″	20170130	Resistant	LC596583	No deletion		911G>A, 1366T>C		649G>A, 1306T>G
*	CCY152	Peru	Lambayeque	S06°30′07″	W79°52′14″	20170131	Resistant	LC596577	No deletion		509A>G, 569A>C, 570G>A, 911G>A	823G>A	727_728insT, 748G>A, 1048T>A, 1306T>G
	CCY154	Peru	Lambayeque	S06°30′07″	W79°52′14″	20170131	Resistant	LC596578	No deletion				1048T>A, 1065G>A, 1306T>G
	CCY156	Peru	Lambayeque	S06°30′05″	W79°52′13″	20170131	Resistant	LC596510	No deletion				1306T>G
	CCY159	Peru	Lambayeque	S06°12′03″	W79°41′56″	20170131	Resistant	LC596556	No deletion		911G>A		1306T>G, 1599C>T
	CCY162	Peru	Lambayeque	S06°12′01″	W79°41′55″	20170131	Resistant	LC596511	No deletion				1306T>G
	CGA028	Peru	Lima	S12°01′35″	W76°41′22″	20161108	Resistant	LC596512	No deletion				1306T>G
	ICA034	Peru	Ica	S13°59′24″	W75°44′31″	20190204	Resistant	LC596564	No deletion				728delT, 1306T>G, 1843A>C
	IND096	Peru	Lima	S12°04′38″	W76°57′00″	20161221	Resistant	LC596529	No deletion				728delT, 1306T>G
	IND103	Peru	Lima	S12°04′42″	W76°57′01″	20161221	Resistant	LC596531	No deletion				728delT, 1306T>G
	IND106	Peru	Lima	S12°04′45″	W76°57′04″	20161221	Resistant	LC596532	No deletion				728delT, 1306T>G
*	JAE035	Peru	Cajamarca	S05°33′13″	W78°50′52″	20190209	Resistant	LC596507	No deletion		911G>A	516G>A	727_728insT, 1306T>G
	JAE036	Peru	Cajamarca	S05°33′15″	W78°51′02″	20190209	Resistant	LC596508	No deletion		911G>A	516G>A	727_728insT, 1306T>G
*	JAE037	Peru	Cajamarca	S05°39′26″	W78°41′28″	20190209	Resistant	LC596509	No deletion		911G>A	516G>A	727_728insT, 1306T>G
	MTP033	Peru	Lambayeque	S06°11′49″	W79°44′28″	20190126	Resistant	LC596558	No deletion				1306T>G, 1599C>T
	PIU029	Peru	Piura	S05°02′33″	W80°34′31″	20190121	Resistant	LC596584	No deletion		1693T>G	1784T>G	1306T>G
	PIU168	Peru	Piura	S05°10′41″	W80°37′00″	20170201	Resistant	LC596522	No deletion		911G>A		1306T>G
	PIU172	Peru	Piura	S05°10′40″	W80°37′02″	20170201	Resistant	LC596557	No deletion		911G>A		1306T>G, 1599C>T
	PIU174	Peru	Piura	S05°10′39″	W80°37′02″	20170201	Resistant	LC596523	No deletion		911G>A		1306T>G
	PKC040	Peru	Lima	S12°10′54″	W76°51′26″	20161119	Resistant	LC596559	No deletion				1306T>G, 1599C>T
	QBB204	Peru	Cusco	S12°54′22″	W72°39′58″	20170226	Resistant	LC596513	No deletion				1306T>G
	QBB215	Peru	Cusco	S12°51′05″	W72°42′02″	20170226	Resistant	LC596514	No deletion				1306T>G
	QBB223	Peru	Cusco	S12°50′46″	W72°42′32″	20170226	Resistant	LC596515	No deletion				1306T>G
	QBB238	Peru	Cusco	S12°50′05″	W72°41′58″	20170226	Resistant	LC596516	No deletion				1306T>G
	SMO068	Peru	Lima	S11°49′14″	W76°21′24″	20161209	Resistant	LC596524	No deletion		911G>A		1306T>G
*	STP088	Peru	Junin	S11°01′21″	W74°58′20″	20161218	Resistant	LC596574	No deletion		911G>A	862G>A	769A>T, 771A>T, 1306T>G
	STP089	Peru	Junin	S11°01′21″	W74°58′21″	20161218	Resistant	LC596582	No deletion		807T>C, 911G>A		1057_1058insC, 1306T>G
	STP090	Peru	Junin	S11°01′22″	W74°58′20″	20161218	Resistant	LC596525	No deletion		911G>A		1306T>G
	STP091	Peru	Junin	S11°01′21″	W74°58′21″	20161218	Resistant	LC596526	No deletion		911G>A		1306T>G
	STP092	Peru	Junin	S11°01′21″	W74°58′21″	20161218	Resistant	LC596527	No deletion		911G>A		1306T>G
	TUM001	Peru	Tumbes	S03°31′51″	W80°13′46″	20181216	Resistant	LC596530	No deletion				728delT, 1306T>G
	TUM004	Peru	Tumbes	S03°31′51″	W80°13′24″	20181216	Resistant	LC596517	No deletion				1306T>G
	TUM007	Peru	Tumbes	S03°31′39″	W80°13′36″	20181216	Resistant	LC596533	No deletion				728delT, 1306T>G
	TUM011	Peru	Tumbes	S03°31′40″	W80°13′46″	20181216	Resistant	LC596534	No deletion				728delT, 1306T>G
	TUM012	Peru	Tumbes	S03°31′25″	W80°13′27″	20181216	Resistant	LC596535	No deletion				728delT, 1306T>G
	TUM015	Peru	Tumbes	S03°32′10″	W80°13′05″	20181216	Resistant	LC596536	No deletion				728delT, 1306T>G
	TUM016	Peru	Tumbes	S03°32′06″	W80°13′07″	20181216	Resistant	LC596573	No deletion				727_728del, 1306T>G
	TUM017	Peru	Tumbes	S03°32′28″	W80°13′02″	20181216	Resistant	LC596537	No deletion				728delT, 1306T>G
	TUM021	Peru	Tumbes	S03°32′27″	W80°12′44″	20181216	Resistant	LC596538	No deletion				728delT, 1306T>G
	TUM023	Peru	Tumbes	S03°32′37″	W80°12′29″	20181216	Resistant	LC596539	No deletion				728delT, 1306T>G
*S. pimpinellifoloum* (*SP*)													
	CCY142	Peru	Lambayeque	S06°44′08″	W79°32′30″	20170130	Resistant	LC596560	No deletion		617G>A		1306T>G, 1599C>T
	CCY164	Peru	Lambayeque	S06°12′02″	W79°41′53″	20170131	Resistant	LC596561	No deletion				1306T>G, 1599C>T
	CGA022	Peru	Lima	S12°01′28″	W76°40′17″	20161108	Resistant	LC596518	No deletion				1306T>G
	CGA026	Peru	Lima	S12°01′35″	W76°40′10″	20161108	Resistant	LC596519	No deletion				1306T>G
	CGA034	Peru	Lima	S12°04′49″	W76°46′10″	20161108	Resistant	LC596540	No deletion				728delT, 1306T>G
*	CPN032	Peru	La Libertad	S07°07′14″	W79°28′06″	20190125	Resistant	LC596528	No deletion		911G>A		1306T>G
	PIU030	Peru	Piura	S04°50′08″	W80°30′35″	20190122	Resistant	LC596562	No deletion				1306T>G, 1599C>T
	PIU031	Peru	Piura	S05°07′09″	W80°11′57″	20190124	Resistant	LC596563	No deletion				1306T>G, 1599C>T
	TUM002	Peru	Tumbes	S03°32′12″	W80°13′30″	20181216	Resistant	LC596541	No deletion				728delT, 1306T>G
	TUM003	Peru	Tumbes	S03°32′12″	W80°13′28″	20181216	Resistant	LC596542	No deletion				728delT, 1306T>G
	TUM005	Peru	Tumbes	S03°31′44″	W80°13′32″	20181216	Resistant	LC596543	No deletion				728delT, 1306T>G
	TUM006	Peru	Tumbes	S03°31′39″	W80°13′37″	20181216	Resistant	LC596544	No deletion				728delT, 1306T>G
	TUM014	Peru	Tumbes	S03°32′07″	W80°13′05″	20181216	Resistant	LC596545	No deletion				728delT, 1306T>G
	TUM018	Peru	Tumbes	S03°32′25″	W80°12′44″	20181216	Resistant	LC596546	No deletion				728delT, 1306T>G
	TUM019	Peru	Tumbes	S03°32′26″	W80°12′43″	20181216	Resistant	LC596547	No deletion				728delT, 1306T>G
	TUM020	Peru	Tumbes	S03°32′27″	W80°12′43″	20181216	Resistant	LC596548	No deletion				728delT, 1306T>G
	TUM022	Peru	Tumbes	S03°32′36″	W80°12′30″	20181216	Resistant	LC596549	No deletion				728delT, 1306T>G
	TUM024	Peru	Tumbes	S03°33′11″	W80°12′24″	20181216	Resistant	LC596550	No deletion				728delT, 1306T>G
	TUM025	Peru	Tumbes	S03°33′11″	W80°12′25″	20181216	Resistant	LC596551	No deletion				728delT, 1306T>G
INIA collection													
*S. lycopersicum* var. *cerasiforme* (*SLC*)													
	PER018795	Peru	Lima	S11°41′69″	W76°52′11″	20150819	Resistant	NT	NT	NT	NT	NT	NT
	PER018836	Peru	Cajamarca	S06°19′12″	W78°41′90″	20111013	Resistant	NT	NT	NT	NT	NT	NT
	PER018878	Peru	Cusco	S12°43′41″	W72°32′44″	20111025	Resistant	NT	NT	NT	NT	NT	NT
	PER018879	Peru	Cusco	S12°41′31″	W72°31′07″	20111025	Resistant	NT	NT	NT	NT	NT	NT
*	PER018894	Peru	Huanuco	S09°50′08″	W76°07′05″	20111109	Susceptible	LC596580	No deletion	854_855del	911G>A		1065G>A, 1306T>G
	PER018901	Peru	Huanuco	S09°48′06″	W76°04′08″	20111110	Resistant	NT	NT	NT	NT	NT	NT
	PER018902	Peru	Huanuco	S09°10′52″	W75°57′36″	20111111	Resistant	NT	NT	NT	NT	NT	NT
	PER018909	Peru	Huanuco	S09°22′55″	W75°01′57″	20111113	Resistant	NT	NT	NT	NT	NT	NT
	PER018923	Peru	Ucayali	S08°23′30″	W75°07′41″	20111116	Resistant	NT	NT	NT	NT	NT	NT
	PER018932	Peru	Ayacucho	S12°54′24″	W74°17′05″	20111213	Resistant	NT	NT	NT	NT	NT	NT
	PER018936	Peru	Ayacucho	S13°03′49″	W73°57′27″	20111214	Resistant	NT	NT	NT	NT	NT	NT
	PER018938	Peru	Ayacucho	S13°06′28″	W73°54′36″	20111214	Resistant	NT	NT	NT	NT	NT	NT
*S. pimpinellifoloum* (*SP*)													
	PER018780	Peru	Lima	S11°02′22″	W77°37′37″	20110816	Resistant	NT	NT	NT	NT	NT	NT
	PER018781	Peru	Lima	S11°02′22″	W77°37′36″	20110816	Resistant	NT	NT	NT	NT	NT	NT
	PER018782	Peru	Lima	S11°01′15″	W77°37′20″	20110816	Resistant	NT	NT	NT	NT	NT	NT
	PER018783	Peru	Lima	S10°59′37″	W77°35′55″	20110816	Resistant	NT	NT	NT	NT	NT	NT
	PER018785	Peru	Lima	S10°39′50″	W77°45′66″	20110816	Resistant	NT	NT	NT	NT	NT	NT
	PER018786	Peru	Lima	S10°39′82″	W77°41′10″	20110817	Resistant	NT	NT	NT	NT	NT	NT
	PER018788	Peru	Lima	S10°40′52″	W77°44′07″	20150817	Resistant	NT	NT	NT	NT	NT	NT
	PER018794	Peru	Lima	S11°29′46″	W76°32′77″	20150817	Resistant	NT	NT	NT	NT	NT	NT
	PER018796	Peru	Lima	S11°29′73″	W77°15′61″	20150819	Resistant	NT	NT	NT	NT	NT	NT
	PER018797	Peru	Lima	S11°29′74″	W77°15′64″	20150819	Resistant	NT	NT	NT	NT	NT	NT
	PER018798	Peru	Lambayeque	S06°27′45″	W79°37′01″	20110914	Resistant	NT	NT	NT	NT	NT	NT
	PER018800	Peru	Lambayeque	S06°26′61″	W79°36′36″	20110914	Resistant	NT	NT	NT	NT	NT	NT
	PER018801	Peru	Lambayeque	S06°26′62″	W79°36′37″	20110914	Resistant	NT	NT	NT	NT	NT	NT
	PER018802	Peru	Lambayeque	S06°25′24″	W79°34′96″	20110914	Resistant	NT	NT	NT	NT	NT	NT
	PER018803	Peru	Lambayeque	S06°25′18″	W79°33′91″	20110914	Resistant	NT	NT	NT	NT	NT	NT
	PER018804	Peru	Lambayeque	S06°20′40″	W79°26′78″	20110914	Resistant	NT	NT	NT	NT	NT	NT
*	PER018805	Peru	Lambayeque	S06°20′38″	W79°26′22″	20110914	Susceptible	LC596576	No deletion	931_932insT	911G>A		1306T>G
	PER018808	Peru	Lambayeque	S06°08′57″	W79°41′69″	20110915	Resistant	NT	NT	NT	NT	NT	NT
	PER018812	Peru	Lambayeque	S06°38′63″	W79°46′34″	20110916	Resistant	NT	NT	NT	NT	NT	NT
	PER018819	Peru	Lambayeque	S06°43′34″	W79°29′20″	20110916	Resistant	NT	NT	NT	NT	NT	NT
	PER018821	Peru	Lambayeque	S06°44′05″	W79°32′96″	20110916	Resistant	NT	NT	NT	NT	NT	NT
	PER018825	Peru	Lima	S11°27′86″	W77°08′14″	20111007	Resistant	NT	NT	NT	NT	NT	NT
	PER018842	Peru	Cajamarca	S05°41′45″	W78°47′78″	20111014	Resistant	NT	NT	NT	NT	NT	NT
	PER018854	Peru	Cajamarca	S05°42′62″	W78°49′46″	20111014	Resistant	NT	NT	NT	NT	NT	NT
	PER018862	Peru	Cajamarca	S05°71′16″	W78°82′40″	20111014	Resistant	NT	NT	NT	NT	NT	NT
	PER018877	Peru	Cusco	S12°43′44″	W72°32′45″	20111025	Resistant	NT	NT	NT	NT	NT	NT
	PER018926	Peru	Ucayali	S08°23′41″	W75°05′32″	20111116	Resistant	NT	NT	NT	NT	NT	NT
	PER018937	Peru	Ayacucho	S13°06′28″	W73°54′37″	20111214	Resistant	NT	NT	NT	NT	NT	NT
	PER018940	Peru	Ayacucho	S13°37′34″	W74°08′44″	20111215	Resistant	NT	NT	NT	NT	NT	NT
TGRC Collection used as references													
*S. lycopersicum* var. *cerasiforme* (*SLC*)													
	LA 1456	Mexico	Veracruz	N19°10′00″	W96°08′00″	1971	Resistant	LC596520	No deletion				1306T>G
	LA 1623	Mexico	Campeche	N20°28′59″	W90°16′59″	19750310	Resistant	LC596569	No deletion				
	LA 1909	Peru	Cusco	S12°51′00″	W72°41′00″	197807	Resistant	LC596521	No deletion				1306T>G
*S. pimpinellifoloum* (*SP*)													
	LA 3123	Ecuador	Santa Cruz Island	S00°37′00″	W90°22′59″	19910516	Resistant	LC596565	No deletion		836A>T		
*S. cheesmaniae* (*SC*)													
	LA 0437	Ecuador	Isabela Island	S00°57′09″	W90°58′39″	19561125	Susceptible	LC596568	400 bp-deletion				
*	LA 0521	Ecuador	Frenandina Island	S00°22′00″	W91°33′00″	1957	Susceptible	LC596567	400 bp-deletion				
*S. galapagense* (*SG*)													
	LA 0438	Ecuador	Isabela Island	S00°58′39″	W91°01′16″	19561126	Susceptible	LC596566	400 bp-deletion				
	LA 0528	Ecuador	Santa Cruz Island	S00°45′00″	W90°19′00″	19570809	Susceptible	LC596571	400 bp-deletion				761G>C, 1306T>G
Commercial cultivars used as reeferences													
*Solanum lycopersicum* var. *lycopersicum* (*SLL*)													
*	cv. Aichi-first (Matsunaga Seed, Konan, Aichi, Japan)						Susceptible	LC596575	No deletion	854_855del	911G>A		1306T>G
*	cv. Momotaro-8 (Takii & Co, Kyoto, Japan)						Resistant	LC596572	No deletion		911G>A		1306T>G

^a^ Date, yymmdd. ^b^ Blank, identical to #AF198177; NT, not tested. ^c^ An approximately 400 bp-deletion including the 5′ UTR and a part of the 5′ ORF of *Asc1* as determined by PCR; * Accessions used in the phylogenetic analyses.

**Table 2:** *Asc1* primers used in this study.

Name	Nucleotide Sequence (5′–3′)	Position ^a^	Tm °C	Thermal Conditions	Reference
Primers to amplify the ca. 1500 bp fragment including *Asc1*					
BASC87	GGAATTCCTGCAATTCATTTGAAACTACAAC	*Eco*R I recognition site + nt 424–447	70	98 °C, 2 min; 30 × (98 °C, 10 s; 59 °C, 30 s; 68 °C, 1 min); 68 °C, 7 min; 4 °C, ∞	Brandwagt et al. (2000)
R12	CAAGTAGTGCTGCCTCTACAAG	nt 2017–1996	61		This study
Primers to detect the ca. 400 bp-deletion in the 5′-UTR and a part of the 5′ ORF of *Asc1* (Figure 1)					
F10	GAAACGATCAAACGTGTT	nt 178–198	56	98 °C, 2 min; 30 × (98 °C, 10 s; 56 °C, 30 s; 72 °C, 1 min); 72 °C, 7 min; 4 °C, ∞	Ago et al. (2016)
R10	CAGGTCCTGCCCAGAAATAC	nt 986–967	63		

^a^ Nucleotide position relative to that of accession #AF198177.

## Supplementary Materials

The following are available online at https://www.mdpi.com/2223-7747/10/1/47/s1, Figure S1: Standardization of pathogenicity in *Alternaria alternata* tomato pathotype As-27 (*Aal*) and leaf necrosis bioassay for AAL-toxin.; Figure S2: Leaflet bioassay of all accessions used in this study for AAL-toxin from a culture extract of *Aal*.; Figure S3: Bayesian inference (BI) tree based on 18 COSII sequences of tomato accessions estimated using MrBayes version 3. 2. 7a [42]; Table S1: COSII nucleotide primers used in this study referred from Rodriguez et al. 2009 [11]; Table S2: GenBank accession numbers of 18 COSII regions nucleotide sequence; Table S3: Model analysis of maximum likelihood (ML) and Bayesian inference (BI).
