# Supplementary material for: Mutations Found in the Asc1 Gene That Confer Susceptibility to the AAL-Toxin in Ancestral Tomatoes from Peru and Mexico
Source: Plants (Basel). 2020 Dec 28;10(1):47. doi: 10.3390/plants10010047 (PMC7824085; doi:10.3390/plants10010047)
Supplement: Supplementary file 1 [file plants-10-00047-s001.zip › Supplemenntary materials/Figure S3.pptx]

## Slide 1
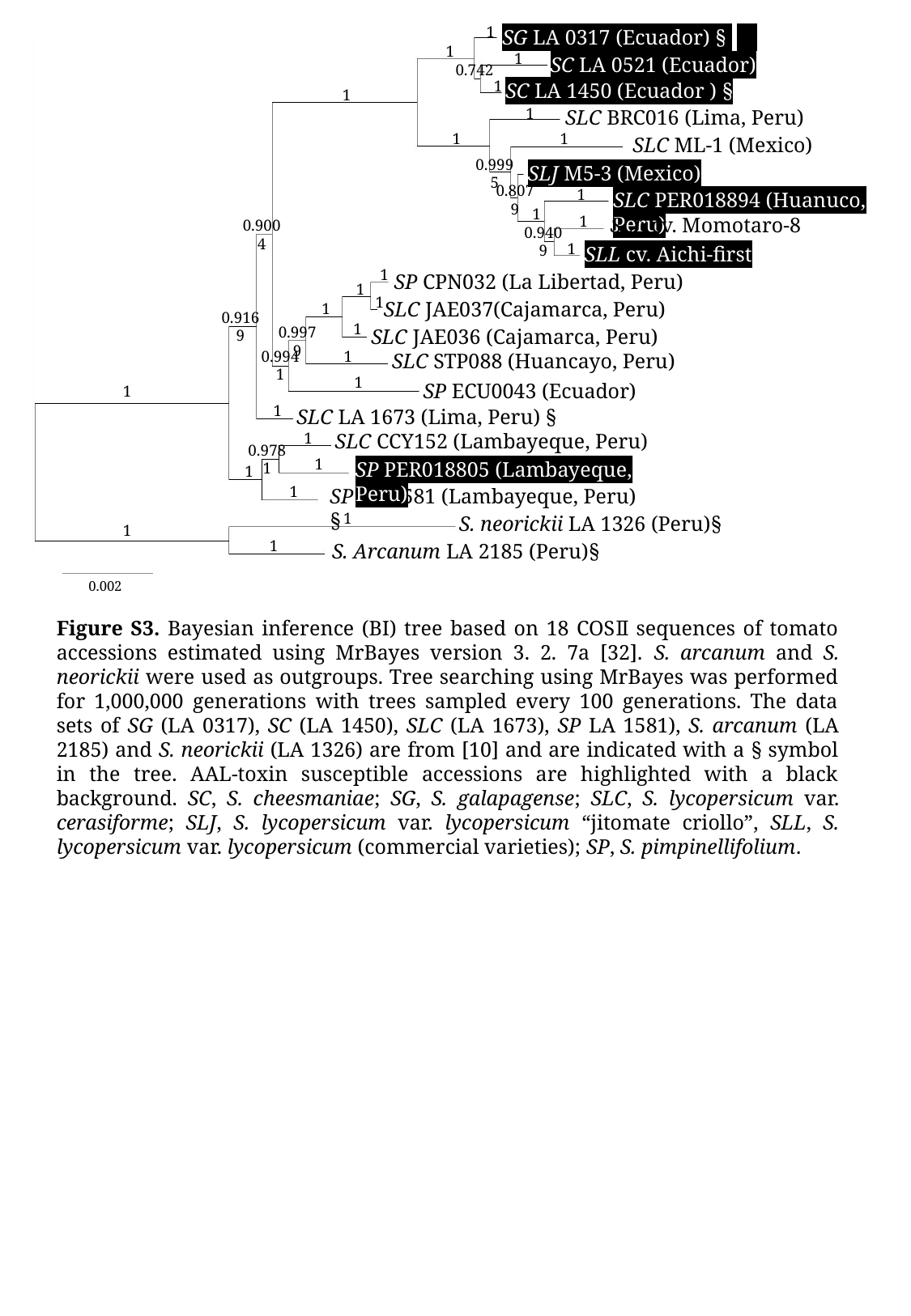

1
SG LA 0317 (Ecuador) §
1
1
SC LA 0521 (Ecuador)
0.742
1
SC LA 1450 (Ecuador ) §
1
1
SLC BRC016 (Lima, Peru)
1
1
SLC ML-1 (Mexico)
0.9995
SLJ M5-3 (Mexico)
0.8079
1
SLC PER018894 (Huanuco, Peru)
1
1
SLL cv. Momotaro-8
0.9004
0.9409
1
SLL cv. Aichi-first
1
SP CPN032 (La Libertad, Peru)
1
1
SLC JAE037(Cajamarca, Peru)
1
0.9169
1
0.9979
SLC JAE036 (Cajamarca, Peru)
0.9941
1
SLC STP088 (Huancayo, Peru)
1
SP ECU0043 (Ecuador)
1
1
SLC LA 1673 (Lima, Peru) §
1
SLC CCY152 (Lambayeque, Peru)
0.9781
1
SP PER018805 (Lambayeque, Peru)
1
1
SP LA 1581 (Lambayeque, Peru) §
1
S. neorickii LA 1326 (Peru)§
1
1
S. Arcanum LA 2185 (Peru)§
0.002
Figure S3. Bayesian inference (BI) tree based on 18 COSⅡ sequences of tomato accessions estimated using MrBayes version 3. 2. 7a [32]. S. arcanum and S. neorickii were used as outgroups. Tree searching using MrBayes was performed for 1,000,000 generations with trees sampled every 100 generations. The data sets of SG (LA 0317), SC (LA 1450), SLC (LA 1673), SP LA 1581), S. arcanum (LA 2185) and S. neorickii (LA 1326) are from [10] and are indicated with a § symbol in the tree. AAL-toxin susceptible accessions are highlighted with a black background. SC, S. cheesmaniae; SG, S. galapagense; SLC, S. lycopersicum var. cerasiforme; SLJ, S. lycopersicum var. lycopersicum “jitomate criollo”, SLL, S. lycopersicum var. lycopersicum (commercial varieties); SP, S. pimpinellifolium.
