# Supplementary material for: Mutations Found in the Asc1 Gene That Confer Susceptibility to the AAL-Toxin in Ancestral Tomatoes from Peru and Mexico
Source: Plants (Basel). 2020 Dec 28;10(1):47. doi: 10.3390/plants10010047 (PMC7824085; doi:10.3390/plants10010047)
Supplement: Supplementary file 1 [file plants-10-00047-s001.zip › Supplemenntary materials/Figure S1.pptx]

## Slide 1
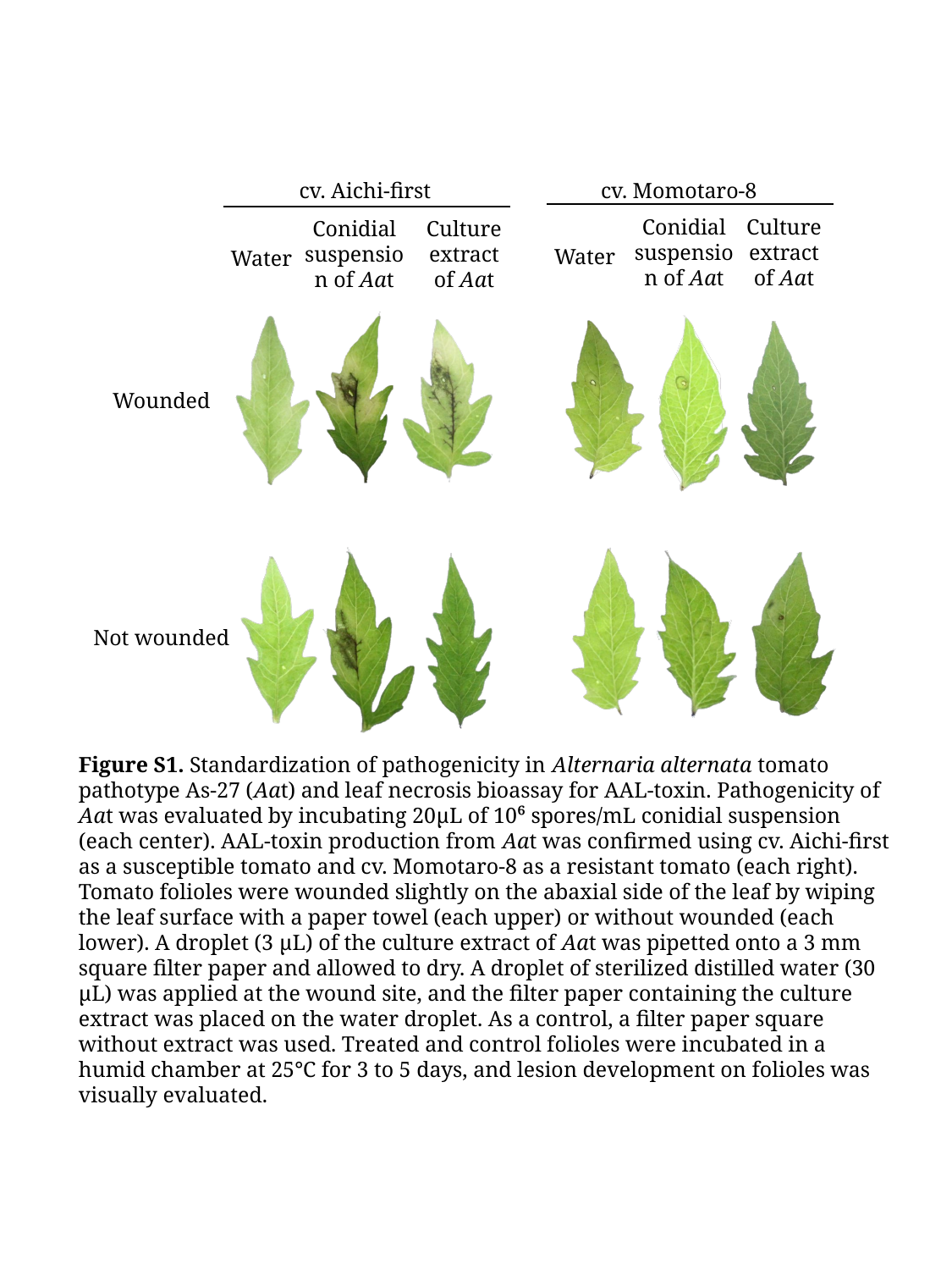

cv. Aichi-first
cv. Momotaro-8
Culture extract of Aat
Conidial suspension of Aat
Water
Culture extract of Aat
Conidial suspension of Aat
Water
Wounded
Not wounded
Figure S1. Standardization of pathogenicity in Alternaria alternata tomato pathotype As-27 (Aat) and leaf necrosis bioassay for AAL-toxin. Pathogenicity of Aat was evaluated by incubating 20μL of 10⁶ spores/mL conidial suspension (each center). AAL-toxin production from Aat was confirmed using cv. Aichi-first as a susceptible tomato and cv. Momotaro-8 as a resistant tomato (each right). Tomato folioles were wounded slightly on the abaxial side of the leaf by wiping the leaf surface with a paper towel (each upper) or without wounded (each lower). A droplet (3 μL) of the culture extract of Aat was pipetted onto a 3 mm square filter paper and allowed to dry. A droplet of sterilized distilled water (30 μL) was applied at the wound site, and the filter paper containing the culture extract was placed on the water droplet. As a control, a filter paper square without extract was used. Treated and control folioles were incubated in a humid chamber at 25℃ for 3 to 5 days, and lesion development on folioles was visually evaluated.
